# Effects of Prenatal Tobacco and Wood-Fuel Smoke Exposure on Birth Weight in Sri Lanka

**DOI:** 10.3390/healthcare5040064

**Published:** 2017-09-26

**Authors:** Malshani L. Pathirathna, Hansani M. Abeywickrama, Kayoko Sekijima, Mieko Sadakata, Naoshi Fujiwara, Yoshiyuki Muramatsu, Kuruppu M. S. Wimalasiri, Upali Jayawardene, Darshana de Silva, Chandraratne M. B. Dematawewa

**Affiliations:** 1 Department of Nursing, Graduate School of Health Sciences, Niigata University, 2-746, Asahimachi-dori, Chuo-ku, Niigata 951-8518, Japan; sekijima@clg.niigata-u.ac.jp (K.S.); atom@clg.niigata-u.ac.jp (M.S.); murayosi@clg.niigata-u.ac.jp (Y.M.); 2 Department of Nursing, Faculty of Allied Health Sciences, University of Peradeniya, Peradeniya 20400, Sri Lanka; hansanimadushika87@gmail.com; 3 Department of Medical Technology, Graduate School of Health Sciences, Niigata University, 2-746, Asahimachi-dori, Chuo-ku, Niigata 951-8518, Japan; fujiwaranaoshi@gmail.com; 4 Faculty of Agriculture, University of Peradeniya, Peradeniya 20400, Sri Lanka; swarnaw@pdn.ac.lk (K.M.S.W.); mahindad@pdn.ac.lk (C.M.B.D.); 5 Teaching Hospital Kurunegala, Colombo Road, Kurunegala 60000, Sri Lanka; upali818@yahoo.com (U.J.); dharshandesilva@yahoo.com (D.d.S.)

**Keywords:** wood fuel smoke, passive smoking, birth weight, Sri Lanka

## Abstract

Low birth weight is a key public health problem in many developing countries, including Sri Lanka. Indoor air pollution from tobacco smoke and kitchen-fuel smoke are among the major contributors to low birth weight, factors of which there are little awareness of in Sri Lanka. We evaluated the effect of passive smoking and kitchen-fuel smoke exposure on birth weight. Seventy-six pregnant women were included in the study. Data were collected by questionnaire, and exposure assessment was conducted using a breath carbon monoxide monitor. Women exposed to second-hand tobacco smoke daily had a significantly lower mean gestational age at delivery (mean ± standard error [SE]: 38.0 ± 0.5 weeks) than women who were exposed to second-hand tobacco smoke only once a week (mean ± SE: 39.3 ± 0.3 weeks) (*p* < 0.05). Women who were exposed to tobacco smoke every day delivered neonates with significantly lower mean birth weight (mean ± SE: 2703 ± 135 g) than women who were only exposed once a week (mean ± SE: 3125 ± 147 g) (*p* < 0.05). A one-minute increase in cooking time in a kitchen without a chimney increased women’s expired air carbon monoxide concentration by 0.038 ppm (*p* = 0.006). Long-term exposure to wood-fuel smoke in a kitchen without a chimney can increase the risk of inhaling high concentrations of carbon monoxide.

## 1. Introduction

Low birth weight (LBW) is defined as body weight less than 2500 g at birth. The World Health Organization estimates that more than 20 million LBW infants are born each year, and that LBW affects approximately 16% of all newborns in developing countries. The prevalence of LBW in Sri Lanka has been 16–17% for many years [1]. LBW is a major cause of infant morbidity and mortality, and developmental disabilities throughout life [2]. However, its rates vary across countries, even within the same region, and depend on socio-economic, behavioral, nutritional, and educational factors. Second-hand tobacco smoke exposure and indoor air pollution remain two major health problems, especially in developing countries, and the effect of passive smoking and cooking smoke on neonatal birth weight has been debated. Smoking during pregnancy can increase the risk of preterm birth, fetal growth restriction, low birth weight, sudden infant death syndrome, and behavioral problems [3,4,5,6]. Although multiple substances have been identified in cigarette smoke, carbon monoxide (CO) and nicotine are the two responsible for adverse effects on the developing fetus [7,8]. CO results from incomplete combustion of biomass such as tobacco or fuel [9]. Once inhaled, CO displaces oxygen to form a stable compound, carboxyhemoglobin (COHb). This reduces the oxygen supply to peripheral tissues and organs, as well as to the fetus [10]. 

Many low- to middle-income countries still use biofuels (wood, agricultural waste, and animal dung) as the main source of energy for cooking and heating. A number of studies have demonstrated a clear association between solid-fuel use and lower respiratory tract infection and chronic obstructive lung diseases [11,12,13]. However, studies on adverse pregnancy outcomes are limited, and few studies have explored the effects of a combined exposure of tobacco smoke and wood-fuel smoke on the developing fetus. 

Sri Lanka is a developing country which has recently been termed a lower- to middle-income country. Wood is the main source of cooking fuel in Sri Lanka, and is often burned over an open fire in an inefficient cooking stove. In addition, liquid petroleum gas and kerosene are used in some households. To our knowledge, this is the first study in Sri Lanka to evaluate the effects of second-hand tobacco smoke and wood-fuel smoke exposure on neonatal birth weight in relation to measured levels of expired CO.

## 2. Materials and Methods

This was part of a larger study carried out to identify maternal factors associated with neonatal birth weight. A prospective study was carried out in a tertiary care hospital in Sri Lanka between October 2015 and June 2016. A more detailed description of the subject recruitment procedure has been published separately [14]. In brief, 150 pregnant women who were between 18–24 weeks of gestation were initially included in the study. Exclusion criteria were risk factors according to obstetrical history (e.g., miscarriages/abortions, multiple fetuses, pregnancy-induced hypertension, and gestational diabetes mellitus) and wider medical history (e.g., psychiatric disorders or long-term cardiac, renal, lung, or gastrointestinal disease).

Eighty-seven pregnant women who visited the hospital antenatal clinic at 30 weeks of gestation were included in this part of the study. Data were collected using a pre-tested questionnaire that included questions on socioeconomic and demographic factors, exposure to tobacco smoke during the pregnancy, the frequency of exposure, relationship to the smoker, use of wood fuel for cooking, time spent cooking, and the characteristics of the kitchen. During the same clinic visit, maternal expired air CO concentrations and percentage of COHb were measured using a piCO^+^ Smokerlyzer breath CO monitor (Bedfont Scientific Ltd., Maidstone, UK). The breath CO monitor is accurate within ±2 ppm and it was calibrated at least every six months in line with the manufacturer’s recommendations. CO and COHb were each measured once only during the clinic visit. Women were asked to hold their breath for 15 s and then blow in to the instrument. Breath CO was measured in parts per million (ppm), and blood COHb was measured as percentage of oxygen replaced. CO levels were defined as equivalent to non-smoker (0–4 ppm), danger zone (5–6 ppm), smoker (7–10 ppm), and frequent smoker (11–16 ppm). The corresponding COHb levels were defined as 0–1.27, 1.43–1.59, 1.75–2.23, and 2.23–3.19%, respectively [15]. The half-life of CO in human blood is approximately 5 h, so measurements indicated recent exposure. Breath CO is not specifically a biomarker of smoking, but it also affected by environmental sources such as motor vehicle exhaust. 

Maternal blood hemoglobin concentrations at the booking visit (weeks 6–8 of gestation) and weeks 28–30 of gestation were obtained from individual pregnancy cards, whereas neonatal birth weight and gestational age at birth were obtained directly from the hospital records after deliveries. Trained research assistants administered the questionnaire and took measurements.

### 2.1. Ethics

The study was approved by the ethical review committees of the Graduate School of Health Sciences at Niigata University, Japan (No. 125), the Faculty of Allied Health Sciences, University of Peradeniya, and the teaching hospital at Kurunegala, Sri Lanka (ERC/2015/06). The study was conducted in compliance with the principles of the declaration of Helsinki. Written informed consent was obtained from the participants. 

### 2.2. Data Analyis

Statistical analysis was carried out using Minitab version 17. Chi-squared test and two-sample *t*-test were used to compare groups exposed and not exposed to tobacco smoke and wood-fuel smoke. Correlations between continuous variables (maternal age, pre-pregnancy BMI, and gestational age at delivery) and neonatal birth weight were evaluated using Pearson’s correlation coefficient. To test the effects of categorical variables (education level, monthly household income, residential area, parity, previous history of LBW, and history of miscarriage and/or abortion) on birth weight, the study used one way ANOVA followed by multiple regression analysis. The variables with *p* < 0.2 in the ANOVA procedure were subsequently included in the multiple regression analysis as dummy variables in addition to the continuous variables mentioned above. Simple regression analysis was used to test the effects of wood-fuel exposure time on mean CO levels. R^2^ (adjusted for the number of predictors in the model) was used to show how much variance is explained by the models. 

At the end of the data collection period, neonatal data were missing for 14 subjects because of ambiguity in hospital delivery registry records, as multiple similar maternal names made it difficult to accurately locate the study participants. The final analysis therefore consisted of maternal-neonatal data for 76 mother–child pairs.

## 3. Results

### 3.1. Characteristics of Participants

Table 1 shows participant characteristics, including smoking exposure and wood-fuel smoke exposure. None of the women were active smokers. Second-hand tobacco smoke exposure was found in 34.2% (26) of the women and 73 women (96.0%) reported that they were exposed to wood-fuel smoke during the pregnancy. Of these 73 women, 12.3% were exposed in a kitchen not equipped with a chimney. Of the women exposed to second-hand tobacco smoke (*n* = 26), 69.2% had been exposed inside the house (closed space) and the others outside. Daily tobacco smoke exposure was reported by 61.5% and 38.5% reported that they were exposed approximately once a week. Of the women exposed to tobacco smoke, 69.2% had been exposed because their husbands smoked and 26.9% because of the father or father-in-law.

### 3.2. Relationship between Maternal Parameters and Birth Weight

Estimates of Person’s correlation coefficient showed that there was a moderate positive correlation between birth weight and gestational age at delivery (r = 0.484; *p* = 0.012). Pre-pregnancy BMI and maternal age had no significant correlation with birth weight (*p* > 0.05).

The fitted multiple regression model showed gestational age at delivery, monthly household income, parity, and previous history of LBW all had a significant impact on neonatal birth weight (*p* < 0.05). Women in the highest income category (≥32,000 LKR) delivered babies with significantly higher mean birth weight than women in the lowest monthly household income category (*p* = 0.013). Mean birth weight of the babies of primiparous mothers was 258 g below those of multiparous mothers (*p* = 0.045) (Table 2).

### 3.3. Exposure Response

Even though it did not reach the level of statistical significance, women who were exposed to daily tobacco smoke had higher levels of expired CO and COHb compared with women who were exposed once a week. Similarly, women exposed to wood-fuel smoke in a kitchen without a chimney had higher levels of expired CO and COHb than women exposed in a kitchen with a chimney (*p* > 0.05). Univariate analysis showed that women exposed to tobacco smoke on a daily basis had a significantly lower gestational age at delivery than women who were exposed once a week (mean ± SE: 38.0 ± 0.5 weeks vs. 39.3 ± 0.3 weeks; 95% confidence interval [CI] for difference: 0.029–2.571; *p* < 0.05). They also delivered babies with significantly lower mean birth weight (mean ± SE: 2703 ± 135 g vs. 3125 ± 147 g; 95% CI for difference: 8–837; *p* < 0.05). When the analysis was restricted to women with a kitchen chimney, the group exposed tobacco-smoke on daily basis still had significantly lower mean neonatal birth weight than the group exposed only once a week (Table 3).

### 3.4. Time Spent Cooking and CO and COHb Measurements

**Figure 1:**
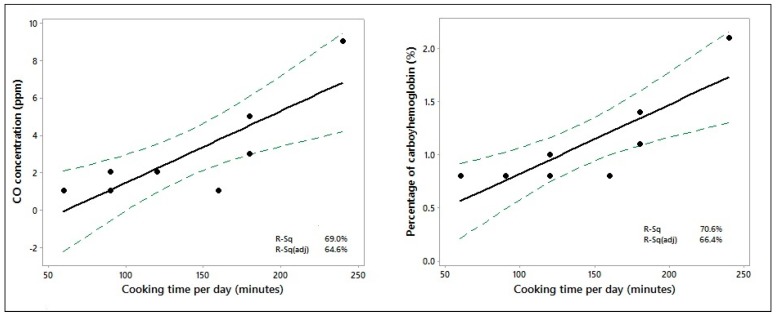
Time spent cooking in a kitchen without a chimney using wood fuel plotted against expired carbon monoxide (CO) concentration and percentage of carboxyhemoglobin (COHb).

There was a weak positive correlation between cooking time and expired CO concentration (r = 0.244; *p* = 0.039) and COHb (r = 0.227; *p* = 0.055) among women exposed to wood-fuel smoke. The correlation was strong when the analysis was restricted to women who were exposed in a kitchen without a chimney (CO: r = 0.831, *p* = 0.006; percent COHb: r = 0.840, *p* = 0.005). Regression analysis found that a 1-min increase in cooking time in a kitchen without a chimney increased expired CO by 0.038 ppm (95% CI: 0.015–0.061; *p* = 0.006) and COHb by 0.006% (95% CI: 0.002–0.010; *p* = 0.005) (Figure 1).

## 4. Discussion

We found that women who were exposed to daily tobacco smoke and women exposed to wood-fuel smoke in a kitchen without a chimney had higher levels of breath CO and COHb. The lack of significance might be because the biomarkers were measured several hours after the women left home. CO has a shorter half-life, so a gap of a few hours from exposure to measurement would reduce the level considerably. Exposure to motor vehicle exhaust fumes on the way to the clinic might also interfere with the estimate of the CO readings. A study in Peru found higher levels of CO in the air in the kitchens of bio-fuel users [16]. However, the shorter half-life of CO in the atmosphere than in the human body makes it difficult to make direct comparisons with our study.

Our findings are based on a small cohort, but this is one of the few prospective studies of which we are aware that assesses personal CO levels during pregnancy against exposure to second-hand tobacco and biomass fuel smoke. None of the women enrolled in our study were active smokers, as hardly any Sri Lankan women smoke. To date, no Sri Lankan studies have been published on second-hand smoke exposure during pregnancy. We found a significantly lower mean birth weight and lower gestational age in babies whose mothers were exposed to tobacco smoke during pregnancy on a daily basis compared with once a week. We also found a moderate positive correlation between gestational age and birth weight. The higher birth weight is probably a result of longer gestation, as the fetus is able to gain additional weight as a result. All the women who were exposed to second-hand tobacco smoke were also exposed to wood-fuel smoke. Second hand tobacco smoke could therefore have a confounding effect on the relationship between wood fuel smoke exposure and birth weight, and wood fuel smoke exposure could act as a confounding variable in the relationship between second hand tobacco smoke exposure and birth weight. It was difficult to test these confounding effects due to difficulty in carrying out a meaningful multiple regression analysis with this smaller sample size. Hackshaw [17] reported that adjusting for several factors can fail the sensible result or they can produce unreliable results when the sample size is small.

A number of studies [16,18,19] have reported reduced mean birth weight and increased risk of LBW with bio-fuel smoke exposure. In Sri Lanka, almost all households use wood as the main source of kitchen fuel, and a small number also use liquid petroleum and kerosene. Expansion of liquid petroleum use is hindered by its high price. Almost all of the women in our study were exposed to wood-fuel smoke, so we were unable to make a fair comparison between those who were exposed and were not exposed to bio-fuel smoke. However, among 12.3% of the women in the study were exposed to wood-fuel smoke in a kitchen without a chimney. The mean birth weight of babies of these mothers was relatively low compared with those whose mothers were exposed to smoke in a kitchen with a chimney (mean ± SE: 2739 ± 184 g vs. 2968 ± 65 g), although the difference was not statistically significant. This lack of significance could however, perhaps be a result of the small sample size and the confounding effect of other variables. We expected to find the highest expired CO concentrations in women exposed to both wood-fuel smoke in a kitchen without a chimney and second-hand tobacco smoke, but the concentrations in this group were lower than in the group exposed to wood-fuel smoke in a kitchen with a chimney and no second-hand tobacco smoke (*p* > 0.05). Longer cooking times may account for the higher expired CO concentrations. In addition, the time since last exposure and the point of data collection is different for each woman may be one of the reasons behind unexpected CO values. 

The lowest birth weight was reported in babies whose mothers cooked in a kitchen without a chimney and were exposed to second-hand tobacco smoke (mean ± SE: 2588 ± 353 g), but the comparison was not statistically significant (*p* > 0.05). 

Gomez et al. [20] reported that expired CO concentrations exceeding 5 ppm in mothers and their spouses were associated with decreased birth weight [19]. In our study, women who were exposed to smoke in a kitchen without a chimney had relatively higher expired CO concentrations, which were close to the upper margin of the non-smoker level. The time spent cooking (using wood fuel) in a kitchen without a chimney was strongly correlated with the levels of expired CO and COHb. We also found that almost all the women without a kitchen chimney had a monthly household income below 32,000 LKR and did not live urban areas. 

A number of possible tactics for reducing bio-fuel smoke exposure have been suggested [13]. These include improved cooking devices, alternative fuel sources, improved living environment, and modifying user behaviors to reduce exposure. Pre-processing (drying) the fuel, good maintenance of stoves, and building stoves at waist level are achievable strategies in Sri Lanka, where the majority of people have a low to medium household income. Using a pot lid while cooking to conserve heat, pre-soaking foods like grains and dhal to reduce cooking time, and not using exhaled breath to start a fire in a wood stove are also simple modifications to user behavior to reduce their exposure to kitchen fuel smoke. However, structural modifications to house (fireplace, chimney, windows, ventilation holes, and separate kitchen) would not be financially feasible in poor community settings. An interventional study, in which efficient stoves were provided to pregnant women in the second and third trimester, found an increase of 89 g in birth weight of babies whose mothers switched from using open fires to stoves [21]. Policymakers could therefore provide cost-effective, efficient stoves to reduce smoke exposure in low-resource communities in Sri Lanka. We found that a considerable proportion of pregnant women were exposed to second-hand tobacco smoke and to wood-fuel smoke. Many households in Sri Lanka still rely on wood for their daily cooking needs, and the women of reproductive age carry a substantial burden of cooking duties, resulting in daily exposure to high concentrations of CO and other air pollutants. 

Provision of antenatal education is recommended, to encourage families to shift toward voluntary smoking restrictions at home and a purposeful reduction of exposure to smoke by pregnant women. Primary health care providers should perform risk assessment, increase awareness, and provide guidance on exposure reduction.

### Limitations

The small cohort is the major limitation of this study. We measured the CO levels during clinic visits by the participants. The time between exposure and measurement may therefore vary from person to person, as could the level of exposure to other sources of CO (such as vehicle exhaust gases) on the way to the clinic, which could affect the accuracy of the CO readings. We did not assess the time gap between the most recent exposure and the point of data collection, and this may cause biased results because of the short half-life of CO. In addition, we were limited to the primary fuel type, although some women may use firewood in combination with liquid petroleum gas and/or kerosene.

Despite the limitations, this is the first study of which we are aware in which the effects of second-hand tobacco smoke and wood-fuel use on neonatal birth weight have been assessed among Sri Lankan women. It is also the first prospective study to our knowledge that provides quantitative exposure response data on neonatal birth weight in relation to measured levels of CO in a Sri Lankan context. Data from this small study can be used to design further large scale studies in Sri Lankan context to assess the effects of smoke exposure on human health. We recommend future studies to assess exposure to smoke among pregnant women and its effects on neonatal birth weight, addressing some of the limitations of this study.

## 5. Conclusions

Second-hand tobacco smoke exposure in Sri Lankan pregnant women is followed by low mean birth weights of their babies. Long-term exposure to wood-fuel smoke in a kitchen without a chimney can increase the risk of inhaling high concentrations of CO.

## Figures and Tables

**Table 1:** Characteristics of the participants.

Variable	All (*n* = 76)	Exposed to Second Hand Tobacco Smoke		Wood Fuel Smoke Exposure		
				Exposed (*n* = 73)		Not Exposed ^a^ *n* = 3
		Yes (*n* = 26, 34.2%)	No (*n* = 50, 65.8%)	In a Kitchen with a Chimney (*n* = 64, 87.7%)	In a Kitchen without a Chimney (*n* = 9, 12.3%)	
Age (years) ^†^	29.3 ± 5.7	27.8 ± 6.4	30.0 ± 5.3	29.7 ± 5.7	26.8 ± 5.3	27.3 ± 6.5
Pre-pregnancy BMI (kg/m^2^) ^†^	22.7 ± 4.3	22.3 ± 4.5	22.9 ± 4.2	22.8 ± 4.3	22.7 ± 4.6	20.0 ± 2.3
Education level, *n* (%)						
Up to primary	9 (12.0%)	4 (16.0%)	5 (10.0%)	8 (12.7%)	1 (11.1%)	-
Secondary/higher	66 (88.0%)	21 (84.0%)	45 (90.0%)	55 (87.3%)	8 (88.9%)	3 (100.0%)
Monthly household income, *n* (%)						
Up to 14,000 LKR	15 (20.0%)	4 (16.0%)	11 (22.0%)	12 (19.0%)	3 (33.3%)	-
14,000 to 32,000 LKR	51 (68.0%)	18 (72.0%)	33 (66.0%)	43 (68.2%)	6 (66.7%)	2 (66.7%)
≥32,000 LKR	9 (12.0%)	3 (12.0%)	6 (12.0%)	8 (12.7%)	-	1 (33.3%)
Residential area, *n* (%)						
Urban	7 (9.3%)	3 (12.0%)	4 (8.0%)	7 (11.1%)	-	-
Sub-urban	35(46.7%)	11 (44.0%)	24 (48.0%)	30 (47.6%)	4 (44.4%)	1 (33.3%)
Rural	33 (44.0%)	11 (44.0%)	22 (44.0%)	26 (41.3%)	5 (55.6%)	2 (66.7%)
Parity, *n* (%)						
Primiparous	23 (30.3%)	7 (26.9%)	16 (32.0%)	15 (23.4%)	5 (55.6%)	3 (100.0%)
Multiparous	53 (69.7%)	19 (73.1%)	34 (68.0%)	49 (76.6%)	4 (44.4%)	-
Previous history of LBW, *n* (%)						
Yes	14 (18.4%)	6 (23.1%)	8 (16.0%)	12 (18.8%)	2 (22.2%)	-
No	62 (81.6%)	20 (76.9%)	42 (84.0%)	52 (81.2%)	7 (77.8%)	3 (100.0%)
History of miscarriage and/or abortion, *n* (%)						
Yes	20 (26.3%)	6 (23.1%)	14 (28.0%)	18 (28.1%)	2 (22.2%)	-
No	56 (73.7%)	20 (76.9%)	36 (72.0%)	46 (71.9%)	7 (77.8%)	3 (100.0%)
Gestational age, weeks ^b,†^	39.1 ± 1.4	38.5 ± 1.8	39.2 ± 1.1	39.1 ± 1.2	38.2 ± 2.4	39.0 ± 1.1
Birth weight, *n* (%)						
<2500 g	13 (17.1%)	5 (19.2%)	8 (16.0%)	10 (15.6%)	2 (22.2%)	1 (33.3%)
≥2500 g	63 (82.9%)	21 (80.8%)	42 (84.0%)	54 (84.4%)	7 (77.8%)	2 (66.7%)
Hemoglobin levels at booking visit (g/dL) ^†^ (*n* = 70)	11.5 ± 1.3	11.4 ± 1.3	11.5 ± 1.2	11.5 ± 1.2	10.8 ± 1.8	11.6 ± 0.4
Anemic at booking visit ^c^, *n* (%)	19 (27.1%)	5 (22.7%)	14 (29.2%)	17 (28.3%)	2 (28.6%)	-
Non-anemic at booking visit ^d^, *n* (%)	51 (72.9%)	17 (77.3%)	34 (70.8%)	43 (71.7%)	5 (71.4%)	3 (100.0%)
Hemoglobin levels at third trimester (g/dL) ^†^ (*n* = 52)	10.9 ± 1.1	10.9 ± 1.1	10.9 ± 1.1	11.0 ± 1.0	10.1 ± 1.4	No data
Anemic at third trimester ^c^, *n* (%)	25 (48.1%)	8 (47.1%)	17 (48.6%)	20 (44.4%)	5 (71.4%)	No data
Non-anemic at third trimester ^d^, *n* (%)	27 (51.9%)	9 (52.9%)	18 (51.4%)	25 (55.6%)	2 (28.6%)	No data

BMI: body mass index; LKR, Sri Lankan rupee; LBW, low birth weight. ^†^ Mean ± SD. ^a^ Women not exposed to wood fuel smoke reported use of liquid petroleum gas for cooking. ^b^ at the time of delivery. ^c^ Hemoglobin < 11g/dL. ^d^ Hemoglobin ≥ 11g/dL.

**Table 2:** Effects of maternal parameters on neonatal birth weight, multiple regression model for neonatal birth weight (g).

Term	Coefficient	95% CI	*t*-Value	*p-*Value
Constant	−2239	−5558–−1080	−1.35	0.183
Continuous variables				
Pre-pregnancy BMI	11.6	−14.0–−37.2	0.91	0.368
Gestational age at delivery	107.3	28.3–186.4	2.71	0.009 **
Categorical variables				
Area of residence (urban)-reference level				
Area of residence (sub-urban)	−50	−436–−335	−0.26	0.795
Area of residence (rural)	241	−145–−627	1.25	0.217
Monthly household income (up to 14,000 LKR)-reference level				
Monthly household income (14,000-32,000 LKR)	204	−75–−483	1.46	0.148
Monthly household income (32,000 LKR)	520	115–926	2.56	0.013 **
Previous history of LBW (yes)-reference level				
Previous history of LBW (no)	311	24–597	2.17	0.034 **
Parity (primiparous)-reference level				
Parity (multiparous)	258	6–510	2.05	0.045 **

R^2^ (adjusted) = 19.26%. *n* = 75. ** *p* < 0.05.

**Table 3:** Exposure to second hand tobacco smoke and wood fuel smoke and their relations to pregnancy outcomes.

				*n*	CO Conc. (ppm)	*p*-Value	COHb (%)	*p*-Value	Cooking Time (min)	*p*-Value	GA (Weeks)	*p*-Value	Birth Weight (g)	*p*-Value
Second-hand tobacco smoke exposure	Yes	All ^a^		26	1.885 (0.178)	0.104 ^a,h^ 0.205 ^b,e^ 0.099 ^c,d^ 0.102 ^c,f^ 0.633 ^d,f^	0.969 (0.049)	0.445 ^a,h^ 0.099 ^b,e^ 0.109 ^c,d^ 0.109 ^c,f^ 0.798 ^d,f^	108 (14)	0.054 ^a,h^ 0.666 ^b,e^ 0.721 ^c,d^ 0.721 ^c,f^ 0.488 ^d,f^	38.5 (0.4)	0.078 ^a,h^ 0.045 ^b,e,**^ 0.523 ^c,d^ 0.523 ^c,f^ 0.367 ^d,f^	2865 (107)	0.408 ^a,h^ 0.046 ^b,e,**^ 0.857 ^c,d^ 0.028 ^c,f,**^ 0.381 ^d,f^
		Daily	All ^b^	16	2.063 (0.232)		1.025 (0.073)		113 (21)		38.0 (0.5)		2703 (135)	
			With kitchen chimney ^c^	13	2.231 (0.257)		1.061 (0.086)		111(25)		38.4 (0.4)		2722 (135)	
			Without kitchen chimney ^d^	3	1.330 (0.333)		0.867 (0.067)		123 (20)		36.6 (2.3)		2617 (497)	
		Once a week	All ^e^	10	1.600 (0.267)		0.880 (0.041)		101 (18)		39.3 (0.3)		3125 (147)	
			With kitchen chimney ^f^	9	1.556 (0.294)		0.889 (0.045)		102 (20)		39.3 (0.3)		3194 (145)	
			Without kitchen chimney ^g^	1	2.000		0.800		90		38.9		2500	
	No	All ^h^		50	2.340 (0.207)		1.015 (0.033)		144 (12)		39.2 (0.1)		2972 (71)	
Wood-fuel smoke exposure	With kitchen chimney		All ^i^	64	2.078 (0.129)	0.379 ^i,l^ 0.476 ^j,k^ 0.323 ^j,l^ 0.244 ^j,m^ 0.111 ^k,m^ 0.262 ^k,n^ 0.158 ^m,n^	0.989 (0.026)	0.614 ^i,l^ 0.991 ^j,k^ 0.638 ^j,l^ 0.086 ^j,m^ 0.071 ^k,m^ 0.359 ^k,n^ 0.190 ^m,n^	130 (10)	0.717 ^i,l^ 0.104 ^j,k^ 0.236 ^j,l^ 0.741 ^j,m^ 0.235 ^k,m^ 0.688 ^k,n^ 0.281 ^m,n^	39.1 (0.2)	0.301 ^i,l^ 0.103 ^j,k^ 0.498 ^j,l^ 0.434 ^j,m^ 0.610 ^k,m^ 0.102 ^k,n^ 0.409 ^m,n^	2968 (65)	0.268 ^i,l^ 0.559 ^j,k^ 0.426 ^j,l^ 0.441 ^j,m^ 0.342 ^k,m^ 0.554 ^k,n^ 0.538 ^m,n^
			Exposed to second-hand tobacco smoke ^j^	22	1.955 (0.203)		0.991 (0.057)		107 (17)		38.8 (0.3)		2915 (109)	
			Not exposed to second-hand tobacco smoke ^k^	42	2.143 (0.165)		0.988 (0.264)		142 (13)		38.2 (0.2)		2995 (81)	
	Without kitchen chimney		All ^l^	9	2.899 (0.873)		1.067 (0.146)		138 (19)		38.2 (0.8)		2739 (184)	
			Exposed to second-hand tobacco smoke ^m^	4	1.500 (0.289)		0.850 (0.050)		115 (17)		37.2 (1.7)		2588 (353)	
			Not exposed to second-hand tobacco smoke ^n^	5	4.000 (1.410)		1.240 (0.242)		156 (31)		38.9 (0.3)		2860 (197)	

CO conc.: carbon monoxide concentration; COHb: carboxyhemoglobin; GA: gestational age; Superscript lowercase letters in *p*-value columns represent the groups used for pairwise comparison. Groups were compared using two-sample *t*-test. Descriptive statistics are expressed as mean (standard error of mean). ** *p* < 0.05.
